# Identification and Visualization of Polystyrene Microplastics/Nanoplastics in Flavored Yogurt by Raman Imaging

**DOI:** 10.3390/toxics12050330

**Published:** 2024-04-30

**Authors:** Xin Ling, Jun Cheng, Weirong Yao, He Qian, Dazhi Ding, Zhilong Yu, Yunfei Xie, Fangwei Yang

**Affiliations:** 1 State Key Laboratory of Food Science and Resources, School of Food Science and Technology, Jiangnan University, Wuxi 214026, China; 6210113056@stu.jiangnan.edu.cn (X.L.); 7220112076@stu.jiangnan.edu.cn (J.C.); yaoweirongcn@jiangnan.edu.cn (W.Y.); qianhe@jiangnan.edu.cn (H.Q.); 8202302013@jiangnan.edu.cn (Z.Y.); 2 School of Food Science and Technology, Jiangnan University, Wuxi 214122, China; 3 School of Microelectronics (School of Integrated Circuits), Nanjing University of Science and Technology, Nanjing 210094, China; dzding@njsut.edu.cn; 4 College of Light Industry and Food Engineering, Nanjing Forestry University, Nanjing 210037, China

**Keywords:** microplastics, polystyrene, food contamination, dietary risk, microscopic Raman imaging, flavored yogurt

## Abstract

The contamination of food by microplastics has garnered widespread attention, particularly concerning the health risks associated with small-sized microplastics. However, detecting these smaller microplastics in food poses challenges attributed to the complexity of food matrices and instrumental and method limitations. Here, we employed Raman imaging for visualization and identification of polystyrene particles synthesized in polymerization reactions, ranging from 400 to 2600 nm. We successfully developed a quantitative model of particle size and concentration for polystyrene, exhibiting excellent fit (R^2^ of 0.9946). We established procedures for spiked flavored yogurt using synthesized polystyrene, providing fresh insights into microplastic extraction efficiency. Recovery rates calculated from models validated the method’s feasibility. In practical applications, the assessment of the size, type, shape, and quantity of microplastics in unspiked flavored yogurt was conducted. The most common polymers found were polystyrene, polypropylene, and polyethylene, with the smallest polystyrene sizes ranging from 1 to 10 μm. Additionally, we conducted exposure assessments of microplastics in branded flavored yogurt. This study established a foundation for developing a universal method to quantify microplastics in food, covering synthesis of standards, method development, validation, and application.

## 1. Introduction

Food packaging, daily necessities, and plastic products have become integral to our daily lives, providing convenience but also generating substantial plastic waste. During the pandemic, the extensive use of plastic items such as takeaway containers and masks has inevitably led to an increase in plastic waste [1,2,3]. These plastic wastes are resistant to natural degradation in the environment, accumulating over time and, under the influence of factors such as UV radiation, hydraulic shear, and biological metabolism, giving rise to numerous microplastic particles [4,5]. This accumulation poses an escalating ecological risk. The concept of “microplastics” is generally defined as plastic particles with a diameter smaller than 5 mm, including fibers, films, and fragments [6]. Correspondingly, nanoplastics are defined as plastics with a size of 1 nm to 1 μm [7]. Recent studies indicate that microplastics, along with adsorbed toxic chemicals, can eventually enter human metabolism through the food chain, affecting human health by disrupting metabolic processes [8,9,10]. Due to the ingestion of food being one of the pathways for human exposure to microplastics, these particles have been identified in human blood, urine, feces, and breast milk in various studies [11,12,13,14]. An increasing number of researchers are investigating the mechanisms through which microplastics may contribute to human diseases. Consequently, their contamination of the food has raised widespread concerns. 

Microplastics have been found in various food items, including seafood, water, soft drinks, milk, honey, edible salt, fruits, vegetables, and meat [15,16,17,18,19,20,21,22]. In a specific study, human body burdens of microplastics through table salt, drinking water, and inhalation were estimated to be (0–7.3) × 10^4^, (0–4.7) × 10^3^, and (0–3.0) × 10^7^ items per person per year, respectively [23]. Yogurt is favored by consumers for its unique taste and rich nutritional content. As of now, there is no research on the release of microplastics from commercially available solid-flavored yogurts, and there is also a lack of established methods for analyzing microplastics in flavored yogurt. Currently, there have been reports on the detection of microplastics in milk and powdered milk [24,25]. People have expressed concerns about the potential ingestion of microplastics through yogurt consumption. Additionally, dairy products such as yogurt or cheese, compared to milk, are exposed to increased pathways for contamination. This study has contributed valuable insights to the research on microplastics in milk products.

Research on the detection of microplastics in complex food matrices typically involves steps such as digestion, density separation, filtration, and identification. Thermogravimetric analysis and spectroscopy techniques are commonly employed for the identification of microplastics. In a research study, researchers presented Py-GC/MS for the detection of microplastics to achieve information for the identification of polymer type and quantification of microplastics based on both particle number and mass of plastics [26]. While these methods can analyze the type and mass of polymers, direct information regarding the characterization of particle size and morphology of microplastics is not provided. To address this limitation, the integration of optical microscopy with Fourier transform infrared spectroscopy (FT-IR) or Raman spectroscopy allows for a more comprehensive analysis of microplastics. The researchers conducted common detection methods involving microscopic counting and the observation of the shape and color of microplastics, coupled with Raman spectroscopy to identify their types [27]. This process is relatively intricate. Various methods for detecting microplastics each have their own advantages and disadvantages, and establishing a rapid and comprehensive detection method is of great significance. In practical applications, spectroscopic techniques are among the most widely used methods. The researchers quantitatively detected polyethylene, polypropylene, polystyrene, and nylon-6 particles in the environment using μ-FTIR, with a detection limit as low as 20 μm [28]. The main problem with FT-IR technology is that the lateral resolution is always limited to a certain diffraction range, so samples that are too small cannot be analyzed (down to 20 μm). Comparatively, Raman spectroscopy exhibits superior lateral resolution (1 μm) compared to FT-IR. By collecting unique spectra with positional information, akin to fingerprints, a thermal map can be generated for the direct visualization and imaging of microplastics [29]. Undoubtedly, imaging techniques based on Raman spectroscopy are superior detection methods.

Raman imaging technology integrates Raman spectroscopy and microscopy, powerful analytical tools that record information carried by monochromatic light from inelastic scattering to study the chemical properties of molecules. This technique is typically non-destructive and non-contact, where each pixel of a chemical image contains molecular information, comprising complete Raman spectra. This allows for enhanced characterization through pseudocolor processing, highlighting the molecular structure and composition of samples, thus improving the precision of feature characterization. Spectral data can be analyzed in various ways to address specific analytical questions, such as enhancing sample characterization by generating false-color images, which more clearly display the chemical structure and composition distribution of samples. In defined test areas, Raman spectra are collected point-by-point at fixed intervals, capturing spatial information. During testing, the laser remains focused on a single sample point, with the sample moving under the laser until the entire area of interest is “imaged”. Peak intensity is a common method for Raman imaging to represent chemical distribution and concentration, and attributes such as multiple peak intensities, peak shifts, peak ratios, and peak widths are also commonly used to generate Raman images for various applications.

Nanoplastics are probably much more dangerous for living organisms than microplastics because they are more reactive [30]. Detecting small-sized microplastics in food has been a challenging research problem. The aim of this study is to utilize microscopic Raman imaging for the visualization and identification of 400–2600 nm polystyrene microplastics. These standards were synthesized through polymerization reactions. A quantitative model of particle size and concentration for polystyrene was established. To validate this technique, we conducted recovery experiments with the pretreatment procedures for yogurt samples. The use of these polystyrene particles would have provided fresh insights into the efficiency of the newly developed microplastic methods of extraction in this study. In real sample detection, we also conducted the assessment of the size, type, and shape of microplastics in flavored yogurt. This study advances exposure assessments of microplastics through branded flavored yogurt.

## 2. Materials and Methods

### 2.1. Materials and Reagents

Sodium dodecyl sulfate, potassium persulfate, anhydrous ethanol, and styrene were purchased from Sinopharm Chemical Reagent Co., Ltd. (Shanghai, China). Polyvinylpyrrolidone was procured from Shanghai Macklin Biochemical Co., Ltd. (Shanghai, China). Azo diisobutyronitrile was obtained from Beijing Bailingwei Technology Co., Ltd. (Beijing, China). Membrane filters (glass fiber microporous membranes, 0.22 µm pore diameter, 50 mm diameter) were purchased from Haiyan New Oriental Plasticizing Technology Co., Ltd. (Jiaxing, China). Multi-enzymatic detergent was purchased from Hangzhou Lvmon Medical Supplies Co., Ltd. (Hangzhou, China). Ethylenediaminetetraacetic acid disodium salt was procured from Beijing Enocare Technology Co., Ltd. (Beijing, China). Tetramethylammonium hydroxide was purchased from Shanghai Titan Scientific Co., Ltd. (Shanghai, China). Flavored yogurts were purchased from a local supermarket in Wuxi.

### 2.2. Preparation and Characterization of Standard Samples of Micro/Nano Polystyrene with Different Particle Sizes

In order to study and evaluate the measurement method, we first prepared polystyrene microplastics with different particle sizes for the establishment of subsequent detection methods. We made modifications to the preparation methods based on the findings from previous studies [31,32]. The specific synthesis steps and reagent formulations are outlined in Table S1. The size, morphology, stability, and dispersion of the synthesized standards were characterized to evaluate the quality of the standards. The particle size distribution, polydispersity index (PDI), and zeta potential of the prepared polystyrene standard were determined using a Malvern laser particle size analyzer. Surface morphology was observed through SEM and TEM. The average diameter, roundness, and percentage of non-spherical particles were analyzed by using ImageJ software (ImageJ version 1.54d, developed by National Institutes of Health, USA, is available at http://imagej.org, accessed on 23 April 2024) to select a certain number of balls on the electron microscope image (Table S2). Thermogravimetric analysis was performed to determine the concentration of the original solution so that the solution of the target concentration could be obtained by dilution. 

### 2.3. Establishment of the Detection Method of Polystyrene Standards for μ-Raman Imaging 

Raman spectra were collected using a Micro Raman Imaging Spectrometer (Thermo Fisher Scientific, Waltham, MA, USA) equipped with a 532 nm laser diode (<10 mW). An EMCCD detector was used to collect Stokes Raman signals under an objective lens (50×) at room temperature (~25 °C) over the wavenumber range of 50–3400 cm^−1^ with an exposure time of 0–5 s for measurements of a single spectrum, or 0.1–100 μm per pixel for imaging. Different Raman detection parameters were set to obtain high signal-to-noise ratio spectra. For Raman imaging of different sizes, polystyrene micro- and nanospheres were scanned over an area of 5 μm × 5 μm with an aperture of pinhole 25 micro. After generating the mapping image, the same position of the same area was scanned again with five different pixel sizes (1, 0.8, 0.6, 0.4, 0.2 μm) to study the effect of the pixel size. 

For Raman imaging of different concentrations, the setting of different concentration standards included the mapping size (80 µm × 40 µm), exposure time (0.3333 s), laser power (6 mW), number of scans (once), lens (50×), and step size (2 µm). The 2 µL solution was absorbed and dripped onto a glass slide covered with tin foil, dried at room temperature, and Raman mapping was performed at the coffee ring. During the evaporation of the solvent, polystyrene migrates from the center of the droplet to its edge, a phenomenon known as the “coffee-ring effect”. It is advantageous for sample preconcentration prior to Raman measurements.

In this study, all Raman mapping images were processed using OMNIC for Dispersive Raman. The Raman signal at 1002 cm^−1^ was picked up to image the polystyrene microscale or nanoscale particles [33]. When a Raman mapping image is generated by Raman intensity at selected characteristic peaks to visualize the plastics, fixed-size acquisition regions produce sets of spectra with different numbers of spectra depending on the step size. That means at any specific position of the Raman mapping image, we can get a full Raman spectrum (including all characteristic peaks ranging from 50 to 3400 cm^−1^) and compare it with the standard spectrum of the reference plastic to distinguish the types of plastic. Directly, when the software comes with a Raman database, it saves the manual comparison step and automatically matches Raman spectra, which is closer to automated detection.

It is crucial to compute an average Raman spectrum for each individual polystyrene pellet to accurately represent its distinctive Raman information. The average spectrum is derived by selecting eight points around the center where the Raman signal is strongest, and the polystyrene of different particle sizes can be visualized in the Raman mapping images. In this study, a series of calibration curves have been developed to investigate the quantitative characterization of particle size or concentration in relation to Raman intensity by utilizing the unique peak associated with polystyrene at 1002 cm^−1^.

### 2.4. Sample Pretreatment Procedures and Extraction of Microplastics from Flavored Yogurt

The sample groups and blank control groups were established. The spiked sample groups were, respectively, set for different particle sizes and concentrations, and the actual sample groups were set without spikes. Each box of yogurt, with a net content of 139 g, was carefully transferred into a glass conical flask and the cup lid and walls were meticulously rinsed. Subsequently, 30 mL of ultrapure water was added. The mixture was then carefully placed in a 40 °C water bath. Following this step, 30 mL of a multi-enzymatic detergent was introduced, and the solution was gently agitated for 2 min. Next, 2.5 g of ethylenediaminetetraacetic acid disodium salt (EDTA-Na_2_) was added, and the mixture was stirred for 3 min. In the next phase, 30 mL of tetramethylammonium hydroxide (TMAH) was carefully added. The solution was then placed in a water bath shaker, operating at a speed of 130 r/min, for a duration of 24 h. Following the completion of the digestion process, the solution was promptly filtered. Posteriorly, the solutions were filtered through in glass fiber microporous membranes (0.22 µm pore diameter, 50 mm diameter) using a suction pump. Then the filter papers were placed in a Petri dish with a cover and dried at room temperature.

### 2.5. Raman Imaging for Identifying Polystyrene Microplastics on Filter Membranes

Using the Raman imaging detection method established in Section 2.3, we conducted Raman imaging on the filter membrane containing extracted polystyrene microplastics. The setting of spiked samples included the mapping size (10 µm × 10 µm), exposure time (0.3333 s), laser power (6 mW), number of scans (once), lens (50×), and step size (0.4 µm). In the absence of spiked samples, the filtered membrane was scanned under a 50× objective of a micro-Raman imaging spectrometer using the “Mosaic” function to detect suspicious particles. After the manual selection of potential polystyrene microplastic candidates, Raman spectral imaging was conducted using a 532 nm laser excitation, an EMCCD detector, and optimized parameters (6 mW laser power, 0.3333 s exposure time, single scan) to achieve high signal-to-noise ratio spectra. 

### 2.6. Determination of Recovery of Microplastics from Spiked Flavored Yogurt

The models derived from the synthesized polystyrene microparticles were used to calculate the recovery rate in the spiked yogurt sample. We added prepared polystyrene standards to actual yogurt samples and measured the concentration values using the previously established quantitative model. The recovery rate of the spiked samples was calculated using Formula (1). This process validated the feasibility of the method. We established procedures for spiked yogurt samples through the use of synthesized polystyrene microparticles, which would have provided fresh insight into the efficiency of the developed microplastic methods of extraction in this study. The final recovery rate indicates an issue, namely the validation of the spiked model established by the standards in the actual yogurt samples. 

For each Raman imaging map of the spiked samples, the average Raman spectrums were calculated. The average Raman intensity at 1002 cm^−1^ was then used to interpolate the concentration from the standard curve, yielding the theoretical value. Recoveries were calculated as follows:

(1) $$\mathrm{Recovery}\left(\%\right){\;=\;C}_{*}{/C}_{0}\;\times \;100$$

where $C_{*}$ was the concentration of polystyrene detected by this method in the spiked samples, and $C_{0}$ was the concentration of the standard polystyrene actually added to the samples.

### 2.7. Risk Assessment

The following equation was used to calculate an individual’s exposure to microplastics by flavored yogurt consumption in accordance with the deterministic model [34]. The estimated daily intake (*EDI*) [35], microplastic contamination factor (*MCF*) [36], microplastic pollution load index (*MPLI*) [36], and polymer risk index (*pR_i_*) [37] in flavored yogurt were calculated using the following formulas, respectively.

(2) $$EDI=\frac{M_{a}\times M_{c}}{B_{w}}$$

*EDI* is estimated daily intake (particles/g bw/day); *M_a_* is the consumption of flavored yogurt (g/day); *M_c_* is the amount of microplastics (particles/g); *B_w_* is body weight (kg) [35]. According to the guidelines issued jointly by the National Health Industry Enterprise Management Association, the Chinese Nutrition Society, the China Dairy Industry Association, and the China Dairy Products Industry Association adults should consume 300 g of dairy products per day. The average body weight of a normal adult is taken as 65 kg.

(3) $$MCF_{i}=\frac{MP_{i}}{MP_{b}}\;$$

(4) $$MPLI=\sqrt[{n}]{MCF_{i}}\;$$

*MP_i_* is the quantity of microplastics in the sample *i*; *n* is the total number of samples. *MP_b_* is the minimum reported average microplastic concentration in processed foods (1.68 particles/kg) [36]. The criteria for the risk category concerning *MPLI* was adopted from Lin et al. [38] (Table 1).

(5) $$pR_{i}=\sum \left(\frac{P_{a}}{P_{t}}\times R_{s}\right)$$

*P_a_* is the quantity of each microplastic polymer in yogurt; *P_t_* is the total number of different microplastic polymers identified in flavored yogurt sample a; *R_s_* is the chemical toxicity coefficient or risk score, such as polystyrene = 30; polyethylene = 11; polypropylene = 1 [39]. The value for the hazard grade and hazard level of each polymer was derived from Lithner et al. [39]. The potential chemical risk of microplastics in flavored yogurt increased with the polymer risk index (Table 1).

### 2.8. Data Analysis

In order to guarantee result reliability, all sample experiments and analyses were conducted in triplicate, utilizing blank samples as controls. The acquired spectral data were analyzed using OMNIC for Dispersive Raman 9.1.24 software (Thermo Fisher Scientific Inc., Waltham, MA, USA). Raman spectra comparisons of suspicious particles were conducted to identify the types of microplastics, utilizing KnowItAll Informatics System 2024 (John Wiley & Sons Inc., Hoboken, NJ, USA). The graphs were plotted using Origin 2019b (Originlab Corporation, Northampton, NC, USA) software. The average diameter, roundness, and percentage of non-spherical particles were analyzed by using ImageJ 1.54d (National Institutes of Health, Bethesda, MD, USA) software to select a certain number of balls on the electron microscope image.

### 2.9. Quality Assurance and Quality Control

To prevent the contamination of microplastics from airborne particles, sampling, laboratory materials, or operator clothing, all experimental manipulations were carried out in a cleanroom under a fume hood. Cotton-based laboratory coats were worn, and a fresh pair of latex gloves was used during each experiment. Additionally, glass instruments were rinsed with ultrapure water before usage, and samples were covered with aluminum foil to minimize exposure to air.

## 3. Results and Discussion

### 3.1. Characterization of Standard Polystyrene with Different Particle Sizes

**Figure 1:**
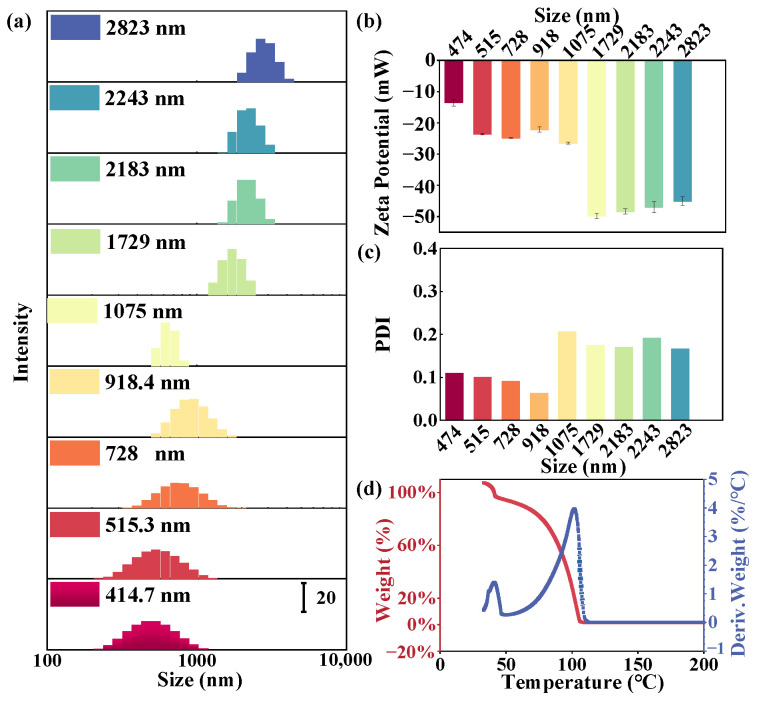
Characterization of prepared polystyrene with different particle sizes. (**a**) Particle size distribution analysis, (**b**) zeta potential, (**c**) PDI, and (**d**) thermogravimetry analysis of 700 nm polystyrene.

We prepared standard polystyrene microplastics with different particle sizes to evaluate the detection methods. The characterization of the synthesized polystyrene was conducted to assess its compliance with standard specifications, as depicted in Figure 1 and Figure S1. In this study, polystyrene microspheres with particle sizes ranging from 400 to 2600 nm were synthesized by controlling the polymerization conditions (Figure 1a).

The zeta potential serves as a metric for the effective electric charge present on the nanoparticle surface. The magnitude of the zeta potential conveys information regarding particle stability, wherein particles with higher magnitude zeta potentials demonstrate increased stability due to elevated electrostatic repulsion between particles. The sign (plus or minus) indicates the nature of the charge on the particle’s surface. All the prepared polystyrene particles exhibit a negative charge, as illustrated in Figure 1b. Notably, as the particle size increases, the absolute value of the zeta potential also increases to nearly 50, leading to heightened stability. Conversely, smaller particle sizes tend to foster precipitation and instability.

PDI is used to estimate the average uniformity of particles in a solution, with larger PDI values indicating a broader size distribution in the particle sample. When the PDI value is less than 0.1, the sample is considered monodisperse. All samples exhibit favorable PDI values (Figure 1c). The TG curve reveals that the overall mass change during this process is 96.58%, resulting in a residue content of 3.42% (Figure 1d and Table S3). The single peak in the curve signifies a weight-loss step primarily attributed to moisture evaporation (Figure S2). The weight loss can be used to determine the moisture content (%) in the polystyrene emulsion. By applying the appropriate formula, the concentration of the 700 nm polystyrene emulsion is calculated to be 34.2 mg/mL.

**Figure 2:**
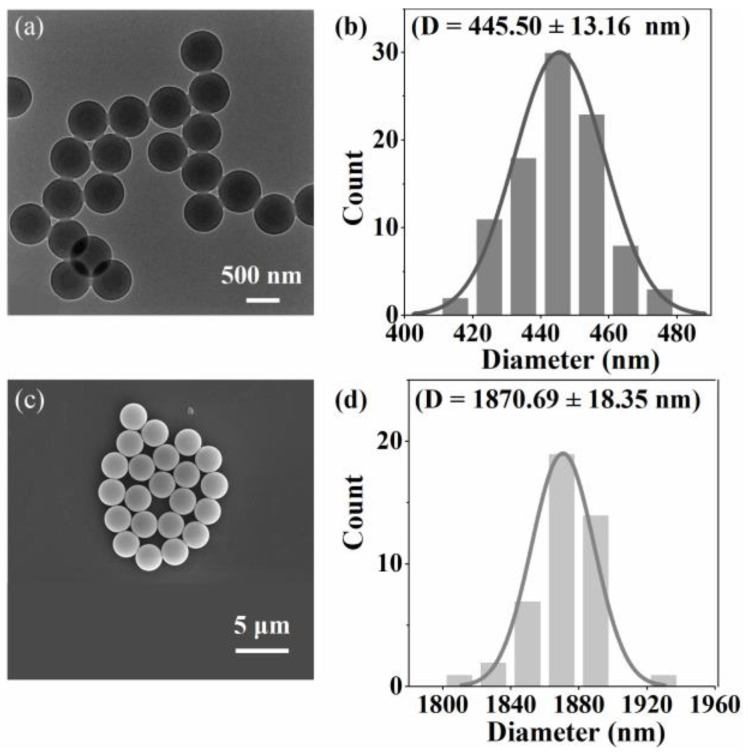
(**a**) TEM image of 515 nm polystyrene; (**c**) SEM images of 1729 nm polystyrene; (**b**,**d**) are grain size distribution histograms of (**a**,**c**), respectively, by ImageJ.

As shown in Figure 2, the surface morphology of polystyrene is highly smooth and exhibits a nearly spherical shape. The size distributions were all unimodal, the polystyrene particles prepared had a roundness of more than 0.94, and no non-spherical particles were observed. Figure 1a and Figure 2b,d represent particle size characterization methods based on different principles. The similarity in their results suggests that the synthesized polystyrene particle size is accurately determined.

Researchers synthesized monodisperse polystyrene spheres in a homogeneous aqueous solution, achieving a controlled size range from 80 nm to 1.65 μm [31,32]. Compared to the characterization results with existing research, we successfully produced stable micro/nanoscale polystyrene spheres, serving as standard samples for Raman detection and model development.

### 3.2. Visualization, Identification, and Quantification of Standard Polystyrene of Different Sizes and Concentration

Before performing Raman imaging on samples, it is customary to establish the optimal parameters for Raman detection to obtain Raman spectra with a high signal-to-noise ratio showed as Figure S3 while balancing measurement time and the resolution of Raman imaging. It has been tested that the spectral signal-to-noise ratio is better at a laser power of 6 mW, exposure time of 0.3333 s, and number of scans of 1 to avoid burning of the sample and test rapidly to save time (Figure S4). 

**Figure 3:**
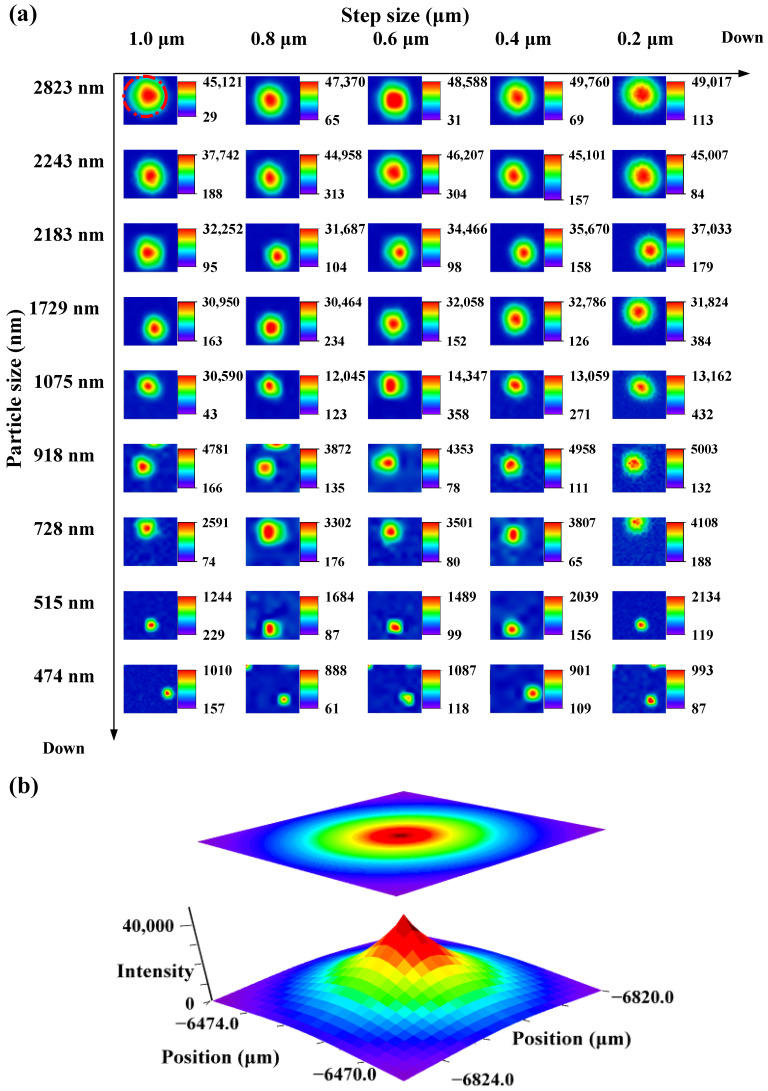
(**a**) Raman mapping 2D image: transverse is associated with different step sizes, while longitudinal pertains to varying particle sizes of single polystyrene particle; (**b**) Raman mapping 3D image of single polystyrene particle.

Due to the intrinsic limitation in the lateral resolution of Raman spectroscopy, which is always confined to a certain diffraction range, microplastics of small sizes are often overlooked. We have investigated the detection limits for particle sizes using Raman imaging methods, as illustrated in Figure 3. Not only can the type of polystyrene be identified from other microplastics by its characteristic Raman peaks, but its approximate size and shape (in this case, regular spherical) can also be determined through visualization methods. Polystyrene particles ranging from 400 to 2800 nm were successfully imaged, with a detection limit of 474 nm. Furthermore, we investigated the impact of varying step sizes on the imaging process. In Raman spectroscopy, the step size is critical for accurate and representative data, representing the interval moved over the sample during data acquisition. Determined by spectral resolution requirements, sample properties, experimental goals, and light intensity, a smaller step size yields more spectra and a higher resolution in Raman mapping but requires longer measurement time. Palonpon et al. explored how step size affects time and space resolution in live cell imaging with Raman microscopy, noting that to maximize optical resolution, the scan step should be at least half the resolution perpendicular to the slit [40]. Imaging of same-size polystyrene micro/nanospheres shows no change with different step sizes at a fixed location. However, adjusting the step size can lead to sample stage drift due to thermal expansion or contraction of mechanical parts, affecting the relative position between the sample stage and the objective lens [41]. Therefore, the positional shift of the spheres in Figure 3a is observed.

**Figure 4:**
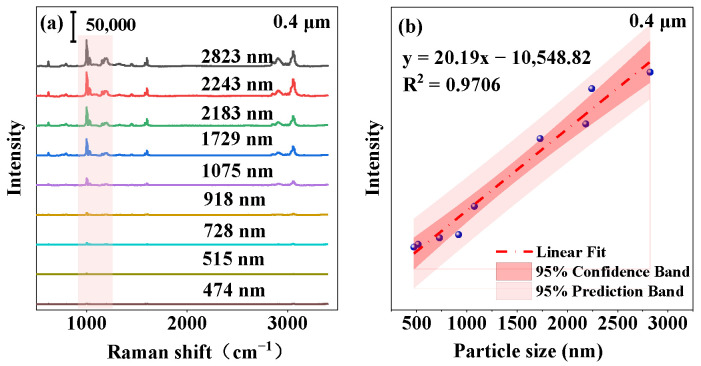
(**a**) Average Raman spectra of polystyrene with different particle sizes at a step size of 0.4 μm; (**b**) standard calibration curve depicting the Raman intensity at 1002 cm^−1^ as a function of particle size.

Under identical step sizes, Figure 4 presents the average Raman spectra of polystyrene across varying particle sizes, showing a clear linear relationship between polystyrene particle size and Raman intensity at 1002 cm^−1^ (assigned to the aromatic ring’s breathing vibration showed in Table S4). The R^2^ values, ranging from 0.9551 to 0.9706 at different step lengths, indicate a robust linear correlation.

**Figure 5:**
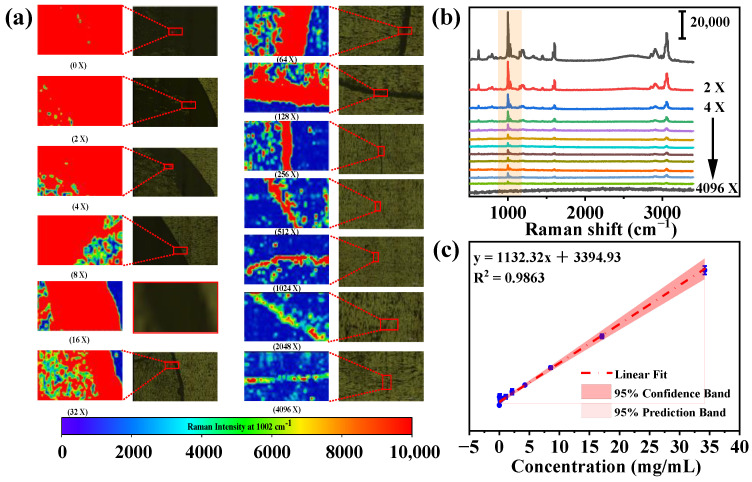
(**a**) Microscopic images and Raman mapping images of 700 nm polystyrene in different concentration ranges, (**b**) average Raman spectrum of 700 nm polystyrene across various concentration ranges using a two-fold dilution method, and (**c**) standard calibration curves of polystyrene with different concentrations.

For samples with low microplastic content, Raman imaging can be applied to individual particles. At higher concentrations, the coffee-ring effect can be utilized for imaging. In actual samples, detection can be facilitated through enrichment and concentration, leading to the formation of a coffee ring. The coffee-ring effect results in a thicker periphery compared to the center of a droplet, caused by solvent evaporation in polystyrene solutions driving capillary flow. This leads to polystyrene molecules migrating outward from the droplet’s core to its rim, culminating in higher polystyrene concentration along the droplet’s edge post-drying [42]. The initial concentration of the 700 nm polystyrene solution, which was 34.2 mg/mL, underwent double dilution using the method of successive dilutions to obtain a solution with varying concentration gradients. Figure 5 shows the optical microscopic and Raman mapping images of the 700 nm polystyrene, and other particle sizes are shown in Figures S5–S7. Upon diluting the original concentration of polystyrene solution by 128 times, the coffee ring remains significantly pronounced. Even at the maximum dilution of 4096 times, with a concentration of 8.3 μg/mL, the observation of the coffee ring becomes challenging, and Raman signals are absent. The average Raman spectra of polystyrene (474 nm, 918 nm, 1729 nm, and 2823 nm) at different concentration showed as Figure S8. The Raman intensity at 1002 cm^−1^ exhibits a strong linear correlation with the concentration (Table S5 and Figure S9). Comparing the LODs (0.1–100 μg/mL) of previously published SERS approaches to our study [43], we do not employ SERS technology, and the lowest detected concentration reached 16.7 μg/mL.

### 3.3. Recovery of Polystyrene from Spiked Flavored Yogurt by Raman Imaging

Successfully recovering polystyrene microplastics from the flavored yogurt matrix is key to effective detection. Flavored yogurt contains essential nutrients such as proteins, fats, and carbohydrates. To avoid interference from organic compounds, sample digestion is necessary before analysis. Common food digestion methods include acid, alkali, and enzymatic digestion [44]. In this study, a combination of enzymes and alkali was employed for sample treatment. The digestion process transforms the milky solid-set yogurt into a dark yellow solution, facilitating subsequent detection and observation after filtration. In this study, disodium ethylenediaminetetraacetate (EDTA) was used as a chelating agent for sodium and calcium particles in yogurt, significantly reducing the formation of calcium-containing soaps during the digestion process and preventing filter clogging. 

**Figure 6:**
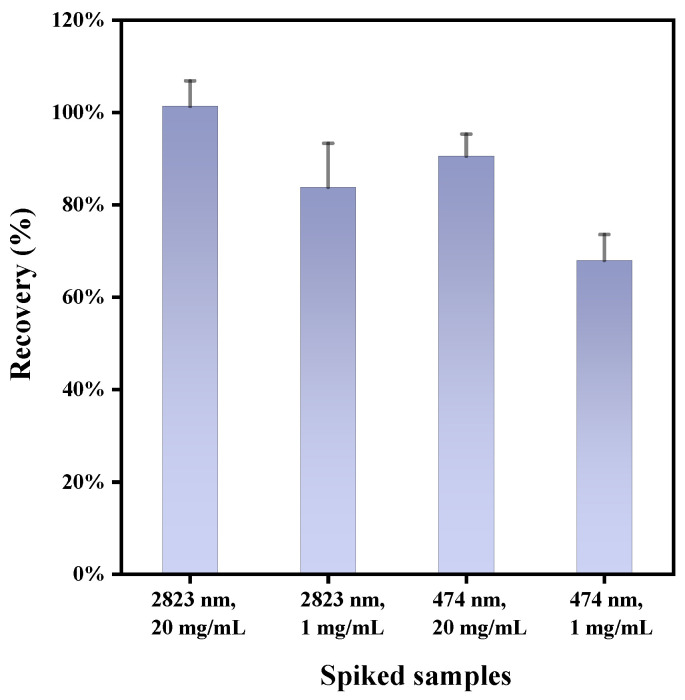
Average recovery rate of the spiked flavored yogurt samples.

Spiking involved testing large-diameter polystyrene (2823 nm) at high and low concentrations (20 mg/mL; 1 mg/mL), and small-diameter polystyrene (474 nm) under similar concentration conditions. By utilizing the developed method for visual and quantitative identification of polystyrene via Raman imaging, concentration detection and recovery rates were assessed. For large particles (2823 nm), average recovery rates were 101.33% (RSD 5.4%) at high concentrations and 83.81% (RSD 11.2%) at low concentrations. For small particles (474 nm), the rates were 90.53% (RSD 5.3%) at high concentrations and 68% (RSD 8.2%) at low concentrations. The variations in recovery rates could be due to calcium-containing soaps forming on filter membranes and the yogurt matrix’s effect. Low-concentration, small polystyrene particles, when dispersed in a large sample (139 g), are difficult to enrich and distribute unevenly on the membrane. Conversely, high-concentration polystyrene forms distinct coffee rings, yielding improved quantification, as depicted in Figure S10. In subsequent work, we synthesized Fe3O4@PEI@Ag nanomagnetic beads to enrich low-concentration polystyrene, addressing the aforementioned limitations. Da Costa Filho employed alkaline digestion and filtration steps, identifying microplastics as small as 5 µm in milk using Raman microscopy and SEM, with recovery rates ranging from 78–141% [45]. In our study, the digestion step was simplified and efficient, yielding favorable recovery rates of 68–101.33% (Figure 6).

### 3.4. Visual Quantification and Raman Spectroscopic Identification of Microplastics in Flavored Yogurt

**Figure 7:**
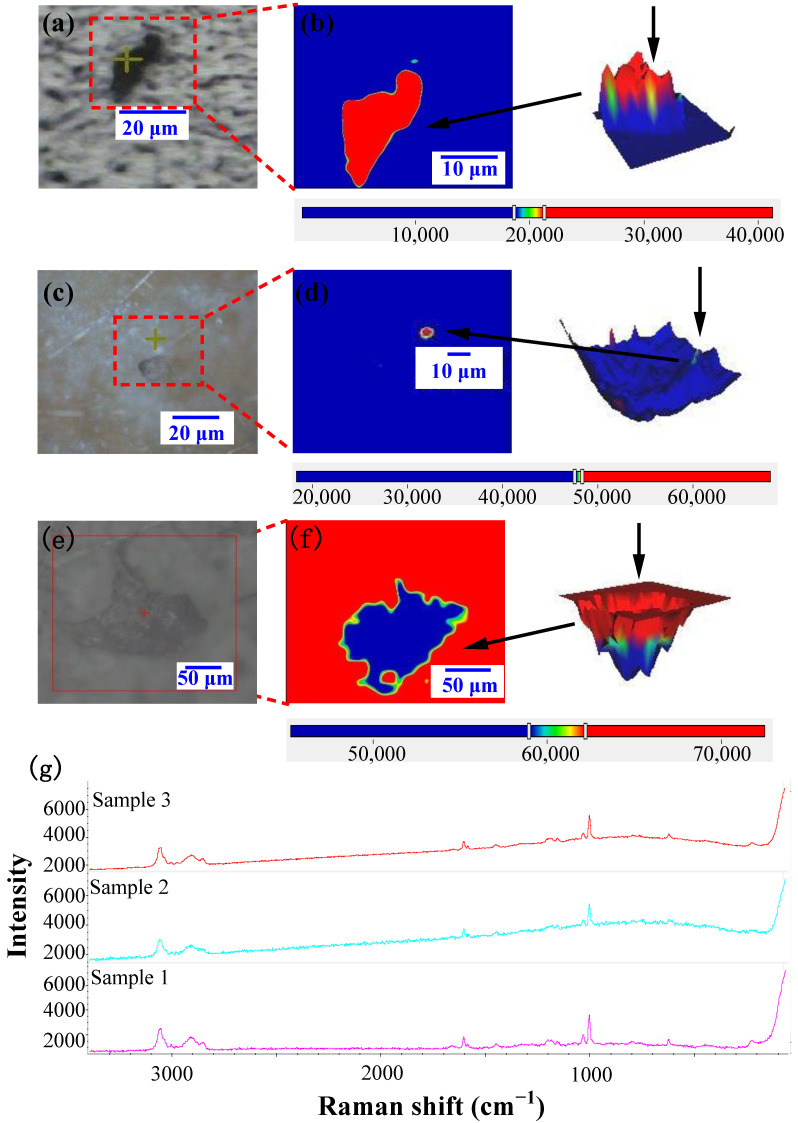
Microscopic images (**a**,**c**,**e**), Raman images (**b**,**d**,**f**), and Raman spectra (**g**) of selected microplastics detected in flavored yogurt.

Representative microplastics were detected in real flavored yogurt samples, exhibiting varying sizes and shapes (Figure 7). Due to the small size of these microplastics, surface color observation is challenging. Raman 2D imaging results were derived from the intensity of polystyrene at 1002 cm^−1^, while 3D Raman imaging combined with microscopic images revealed predominantly irregularly shaped polystyrene particles. Employing both single and multiple-component analyses, as well as functional group analyses, the acquired spectra were systematically compared against a comprehensive Raman database. The most suitable match was identified based on high-score matches and corresponding peak positions. The matching identified various types of microplastics, including polystyrene, polypropylene, and polyethylene, with matching coefficients exceeding 70 (Figure S11). The potential sources of strong peaks in the 1300–1700 cm^−1^ range for polyethylene may include other synthetic polymers and molecules present in yogurt. This indicates that yogurt packaging is not the sole source of contamination. The detected microplastics may originate from flavored yogurt raw materials, factory production processes, and the environment.

Our detection methodology and data have been systematically compared with those of other dairy products, such as milk. Gurusamy’s research team detected a low level of microplastics (3–11 particles/L) in branded milk [27]. The researchers employed a pretreatment method involving milk heating to prevent filter clogging. However, the use of an 11 μm filter aperture led to an underestimation of small-sized microplastics. Raman identification revealed that polyethersulfone and polysulfone are common in milk samples, and also prevalent as membrane materials in the dairy production process. In another study, microplastics were detected in milk using a heating and filtration method, with ATR-FTIR employed for analysis. The average microplastic concentration in all milk samples ranged from 1 to 16 particles/L, and the smallest particle size analyzed by FT-IR was 25 µm. The study identified five different polymer types, namely nylon-6, polyethylene terephthalate, ethylene vinyl acetate, polypropylene, and polyurethane, in the analyzed packaged milk samples [35]. 

**Figure 8:**
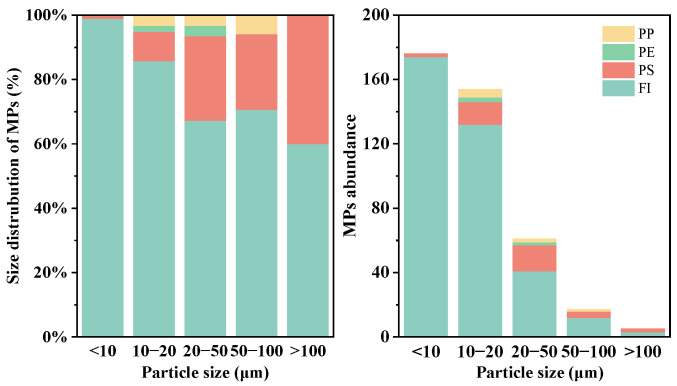
The size distribution and abundance of detected microplastics from three flavored yogurt samples. FI means microplastics that failed to be detected.

The types and size distribution of microplastics extracted from yogurt samples are illustrated in Figure 8. No microplastics were detected in the blank control group, while all actual samples showed the presence of microplastics. The identified microplastic types were primarily polystyrene, polypropylene, and polyethylene, constituting approximately 20%, 3%, and 1%, respectively. About 76% of particles did not exhibit Raman spectra. The yogurt packaging materials include polystyrene for the cup wall and polypropylene for the plastic film, suggesting that food packaging may be a source of microplastics. However, the traceability of microplastics is a complex process related to yogurt production and packaging. Most of the extracted particles, which were of uncertain microplastic identity, exhibited sizes smaller than 20 μm, particularly those under 10 μm. Among particles in the 10–20 μm range, only 14% were confirmed as microplastics, and for particles smaller than 10 μm, only about 1–2% were detected as polystyrene. Some particles were observed under the microscope without Raman characteristic peaks, labeled as FI. The correlation between the proportion of FI microplastics and size is evident in Figure 8. Smaller-sized examples often lack distinct characteristic peaks. The detection of small-sized microplastics using Raman spectroscopy in certain food items has already been demonstrated. For instance, microplastics as small as 1.5 μm in mineral bottled water were identified [46]. In more complex food matrices, microplastics as small as 1 μm were identified in edible oil [47]. While these two studies focused on a simple liquid matrix, in our study, we are able to detect microplastics in the range of 1–10 μm from gelled-flavor yogurt. Overall, smaller particles are more challenging to identify, influenced not only by instrument detection limits but also by food matrix pre-processing methods. For instance, during sample filtration, if the selected membrane pore size is larger than the microplastic particle size, there may be a loss of microplastics and, consequently, lower detection results. The filter membrane used in this study had a pore diameter of 0.22 μm, which should capture microplastics larger than this size. Additionally, factors such as aging or pigment coverage of microplastics can affect detection results. This is also key to overcoming challenges in detecting small sizes in the future.

Utilizing an efficient combination of enzymatic and alkaline digestion, we successfully separated sub-10 μm polystyrene microplastics from real yogurt samples and confirmed their presence through Raman imaging, establishing the method’s viability. However, due to the low abundance of microplastics in actual samples, quantification was less than ideal, suggesting its greater applicability to samples with higher microplastic concentrations. Alternatively, future improvements could be made with better enrichment techniques.

### 3.5. Risk Assessment

According to the “Guidelines for Dairy and Dairy Product Consumption among Chinese Residents”, adults are recommended to consume 300 g of dairy products daily, with an estimated daily intake (EDI) for adults at 0.56 micrograms per gram of body weight per day. During fermentation, yogurt undergoes nutritional changes due to the hydrolysis of lactose, proteins, and fats, enhancing its digestibility and absorption. This process particularly benefits the elderly or those with lactose intolerance by reducing or eliminating the occurrence of lactose intolerance symptoms. For children in rapid growth stages, a higher intake of quality proteins and calcium is essential. It is advised that preschool children aged 2–5 years consume 350–500 g of yogurt daily. Consequently, in certain groups, the recommended yogurt intake may exceed 300 g, potentially increasing the risk of microplastic ingestion.

The microplastic contamination factor (MCF) and microplastic pollution load index (MPLI) in flavored yogurt are identified as 10 and 2.15, respectively, indicating a medium level of contamination and classifying the risk as Category I. Lin and colleagues, using information from globally published studies [38], determined the MPLI in cow’s milk to be 22.8, categorizing it as a risk level of Category III. Food items such as table salt, sugar, and bottled water have been identified as belonging to risk Category II, while processed meats, canned fish, honey, beer, and vinegar fall under risk Category I (Table 1).

The polymer risk index (pR_i_) for flavored yogurt is marked at 16, indicative of a low hazard level. Conversely, Lin et al. computed a pR_i_ of 337 for cow’s milk, categorizing its risk as moderate [38]. This elevated risk in cow’s milk relative to yogurt may stem from a greater diversity of exposure routes, resulting in heightened microplastic contamination. Comparative studies of microplastic abundance in dairy products have shown that cow’s milk possesses a higher microplastic concentration than flavored yogurt. Further, in the same studies, items such as canned fish, beverages, and table salt exhibited pR_i_ values surpassing that of cow’s milk, at 1032, 823, and 766, respectively [38]. The pR_i_ for tap water stands at 290 [48], with table salt at 182.

## 4. Conclusions

In this study, we successfully identified and visualized individual microplastics in the range of 400–2600 nm. Utilizing microscopic Raman imaging, we established a quantitative model for the size and concentration of microplastics. To validate this technique, we conducted recovery experiments with yogurt sample pretreatments, achieving recovery rates of 68–101% for corresponding particle sizes and concentrations. The use of these polystyrene particles would have provided fresh insights into the efficiency of the newly developed microplastic methods of extraction in this study. Additionally, we applied this Raman imaging technique to analyze real flavored yogurt samples to conduct the assessment of the size, type, and shape of microplastics. This study advances exposure assessments of microplastics through flavored yogurt.

These findings are promising, as the research effectively balances imaging resolution and detection time, limiting the imaging time for individual microplastics to a few minutes. However, a limitation of the study is that despite imaging standard microplastics as low as 400 nm, particles detected and visualized in actual samples were minimal at 1–10 μm. Notably, 76% of suspicious particles in the detection results were not identified as microplastics, with 48% having diameters below 10 μm. These unidentified particles may not be plastic or could fail identification due to factors such as size, shape, pigment coverage, or aging. The identification accuracy of Raman spectroscopy for plastic particles smaller than 1 μm will be enhanced through further research on factors influencing Raman characteristics.

## Figures and Tables

**Table 1:** Risk-level criteria for chemical risk and pollution load of microplastics [38].

Polymer Risk Index	Hazard Level	Pollution Load Index	Risk Category
Close to 1	Very low hazard	<10	I
Close to 10	Low hazard	10–20	II
Close to 100	Medium hazard	20–30	III
Close to 1000	High hazard	>30	IV
Close to 10,000	Very high hazard	−	−

“−”: The highest risk category for PLI was defined as IV.

## Data Availability

The original contributions presented in the study are included in the article/Supplementary Materials, further inquiries can be directed to the corresponding author.

## Supplementary Materials

The following supporting information can be downloaded at: https://www.mdpi.com/article/10.3390/toxics12050330/s1. Table S1: Synthesis formula; Table S2: Mean diameter, area, roundness of the prepared particles, and the percentage of non-spherical particles; Table S3: TG-DSC analysis of different particle size of polystyrene; Table S4: Wavenumber (cm^−1^) and band assignment of Raman spectra of polystyrene; Table S5: Standard calibration curves and R2 values for polystyrene at different concentrations; Figure S1: (a) TEM image of 515 nm polystyrene; (b–f) SEM images of 474 nm, 728 nm, 918 nm, 1729 nm, and 2823 nm polystyrene; (g–l) Grain size distribution histogram of (a–f) by ImageJ; Figure S2: TG graph, DTG graph, and DSC graph of polystyrene: (a) 474 nm polystyrene; (b) 918 nm polystyrene; (c) 1729 nm polystyrene; (d) 2823 nm polystyrene; Figure S3: Raman spectra analysis of polystyrene; Figure S4: (a) Raman spectral signal-to-noise ratio at different laser powers; (b) Raman spectral signal-to-noise ratios at different exposure times; Figure S5: Raman mapping and microscopy images of polystyrene at 474 nm under various dilution factors; Figure S6: Raman mapping and microscopy images of polystyrene at 1729 nm under various dilution factors; Figure S7: Raman mapping and microscopy images of polystyrene at 2823 nm under various dilution factors; Figure S8: The average Raman spectra of polystyrene (474 nm, 918 nm, 1729 nm, and 2823 nm) at different concentrations; Figure S9: The calibration curves of polystyrene (474 nm, 918 nm, 1729 nm, and 2823 nm) at different concentrations; Figure S10: Coffee ring in the spiked flavored yogurt; Figure S11: A comparison between obtained spectra and standard polymer spectra.
