# Supplementary material for: Identification and Visualization of Polystyrene Microplastics/Nanoplastics in Flavored Yogurt by Raman Imaging
Source: Toxics. 2024 Apr 30;12(5):330. doi: 10.3390/toxics12050330 (PMC11125995; doi:10.3390/toxics12050330)
Supplement: Supplementary file 1 [file toxics-12-00330-s001.zip › toxics-2997163-supplementary.pdf]

Supporting Information

Table S1 Synthesis formula

Table S2 Mean diameter, area, roundness of the prepared particles, and the percentage of non-spherical particles

Table S3 TG-DSC analysis of different particle size of polystyrene

Table S4 Wavenumber(cm^{-1}) and band assignment of Raman spectra of polystyrene

Table S5 Standard calibration curves and R^2 values for polystyrene at different concentrations

Figure S1 (a) TEM image of 515 nm polystyrene; (b)-(f) SEM images of 474 nm, 728 nm, 918 nm, 1729 nm, 2823 nm polystyrene; (g)-(l) Grain size distribution histogram of (a)-(f) by ImageJ.

Figure S2 TG graph, DTG graph, DSC graph of polystyrene: (a) 474 nm polystyrene; (b) 918 nm polystyrene; (c) 1729 nm polystyrene; (d) 2823 nm polystyrene

Figure S3 Raman spectra analysis of polystyrene

Figure S4 (a) Raman spectral signal-to-noise ratio at different laser powers; (b) Raman spectral signal-to-noise ratios at different exposure times

Figure S5 Raman mapping and microscopy images of polystyrene at 474 nm under various dilution factors

Figure S6 Raman mapping and microscopy images of polystyrene at 1729 nm under various dilution factors

Figure S7 Raman mapping and microscopy images of polystyrene at 2823 nm under various dilution factors

Figure S8 The average Raman spectra of polystyrene (474 nm, 918 nm, 1729 nm, and 2823 nm) at different concentration

Figure S9 The calibration curves of polystyrene (474 nm, 918 nm, 1729 nm, and 2823 nm) at different concentrations

Figure S10 Coffee ring in the spiked flavored yogurt.

Figure S11 A comparison between obtained spectra and standard polymer spectra.

Preparation and characterization of standard samples of micro/nano polystyrene with different particle sizes

We made modifications to the preparation methods based on the findings from previous studies [1, 2]. Preparation of micro/nano polystyrene standard spheres were conducted in a 500 mL four-necked flask equipped with a constant-pressure dropping funnel, nitrogen gas inlet and outlet, placed in a constant-temperature oil bath on a heat-collecting magnetic stirrer. The detailed operational steps were as follows: Firstly, specified amount of initiator (AIBN or $K_2S_2O_8$), emulsifier (PVP or SDS), and dispersant (anhydrous ethanol) were added into the four-necked flask. Nitrogen gas was flowed through the gas inlet for half an hour to eliminate oxygen from the solution in the four-necked flask. Simultaneously, temperature of the heat-collecting constant-temperature magnetic stirrer was raised to a predetermined level. Subsequently, nitrogen gas was introduced into the constant-pressure dropping funnel via a gas inlet for ten minutes to remove oxygen from the styrene solution. Then, styrene was dropped at a constant rate while stirring at a consistent speed. Allowed the reaction to proceed for a specific duration. The specific reagent formulations are outlined in Table S1.

Table S1 Synthesis formula

	Emulsifier		Initiator		monomer	Dispersant	Temperature	Rate	Time
	PVP(g)	SDS(g)	AIBN(g)	$K_2S_2O_8$(g)	Styrene(mL)	C_2H_6O(mL)	T (℃)	v(r/min)	t(h)
dispersion polymerization	5		1		55	600	65	1200	16
	5		1		40	300	65	1200	16
	5		1		30	300	65	1200	16
	5		1		40	300(3:1)	65	1200	16
	10		1		40	300	65	1200	16
emulsion polymerization		0.033		0.1	1.50	70	70	120	8
		0.066		0.1	3.00	70	70	120	8
		0.100		0.1	4.50	70	70	120	8
		0.120		0.1	5.25	70	70	120	8
		0.140		0.1	6.50	70	70	120	8

Roundness is a quantitative shape factor, calculated as follows:

$$Roundness=\frac{4}{\pi}\times\frac{A}{D^2}$$

Where *A* represents the area and *D* denotes the diameter. The value of roundness ranges from 0 to 1, where 1 signifies a perfect circle. In this study, spherical particles are defined as those with a roundness $\geqslant 0.75$, while non-spherical particles are defined as those with a roundness <0.75.

Using image analysis software (ImageJ version 1.53c, developed by NIH, available at http://imagej.nih.gov/ij/), a certain number of spheres were selected from the electron microscopy images of each particle size. Further analyses were conducted to determine the average diameter, roundness, and percentage of non-spherical particles of the polystyrene sphere standards of different particle sizes.

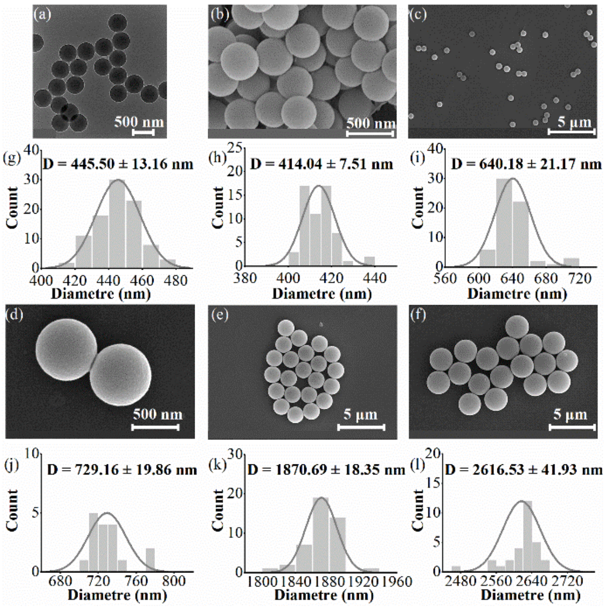

Figure S1 (a) TEM image of 515 nm polystyrene; (b)-(f) SEM images of 474 nm, 728 nm, 918 nm, 1729 nm, 2823 nm polystyrene; (g)-(l) Grain size distribution histogram of (a)-(f) by ImageJ.

Table S2 Mean diameter, area, roundness of the prepared particles, and the percentage of non-spherical particles

Size(nm)	Number	Diameter(nm)	Area(nm^2)	Roundness	non-spherical particles (%)
474	57	414.04	128614.90	0.96	n.d.
515	96	445.50	153859.93	0.99	n.d.
728	64	640.18	304713.91	0.95	n.d.
918	17	729.16	408153.71	0.98	n.d.
1729	44	1870.69	2695593.94	0.98	n.d.
2823	29	2616.53	5242088.54	0.97	n.d.

Note: n.d.: not detection.

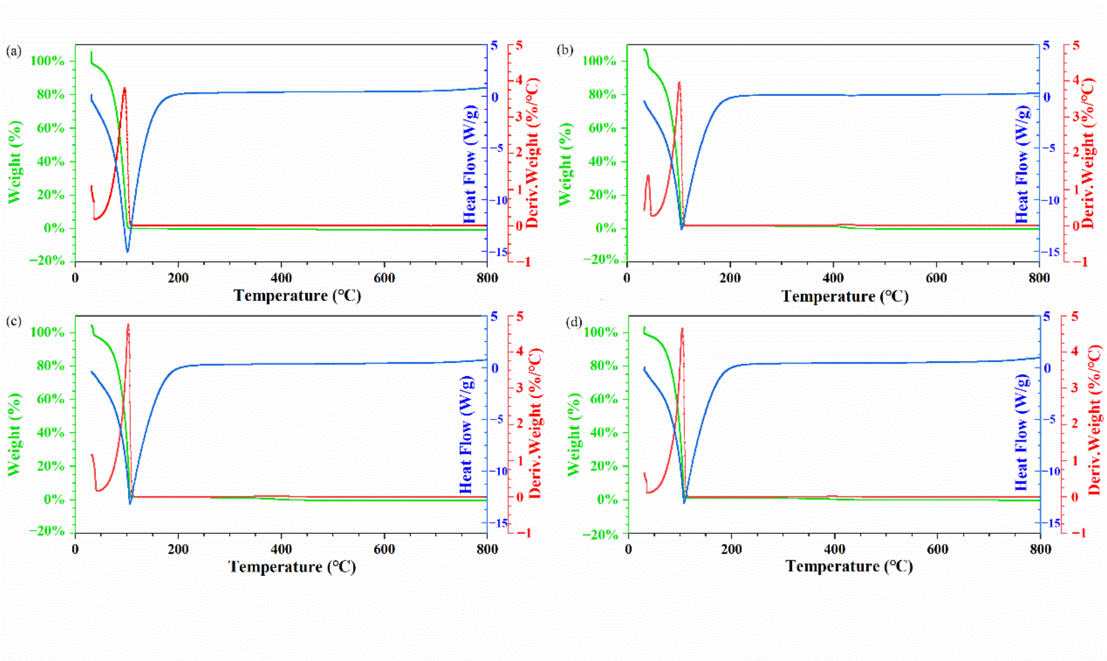

Figure S2 TG graph, DTG graph, DSC graph of polystyrene: (a) 474 nm polystyrene; (b) 918 nm polystyrene; (c) 1729 nm polystyrene; (d) 2823 nm polystyrene

Table S3 TG-DSC analysis of different particle size of polystyrene

Size(nm)	Initial reaction temperature (℃)	Temperature of maximum reaction rate (℃)	Reaction termination temperature (℃)	Maximum reaction rate (%/℃)	Residue content (%)	Polystyrene emulsion content (mg/mL)
474	78.87	101.11	96.26	3.802	2.14	21.4
918	86.67	101.43	106.11	3.940	3.42	34.2
1729	88.75	102.72	106.28	4.717	3.36	33.6
2823	89.19	104.65	107.80	4.650	2.82	28.2

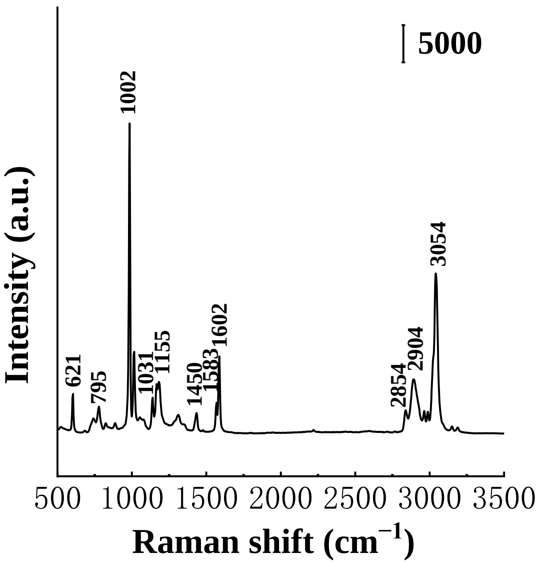

Figure S3 Raman spectra analysis of polystyrene

Table S4 Wavenumber(cm^{-1}) and band assignment of Raman spectra of polystyrene

Number	Band (cm^{-1})	Assignment	References
1	621	Ring deformation mode	[3]
2	795	C-H out-of-plane deformation mode	[3]
3	1002	Ring breathing mode	[3]
4	1031	C-H in-plane deformation mode	[3]
5	1155	C-C stretch mode	[3]
6	1450	CH2 scissoring mode	[3]
7	1583	C=C stretch mode	[3]
8	1602	Ring skeletal stretch mode	[3]
9	2854	CH_2 aliphatic stretch mode symmetric	[3]
10	2904	CH_2 aliphatic stretch mode antisymmetric	[3]
11	3054	C-H bonds stretching on the benzene ring	[3]

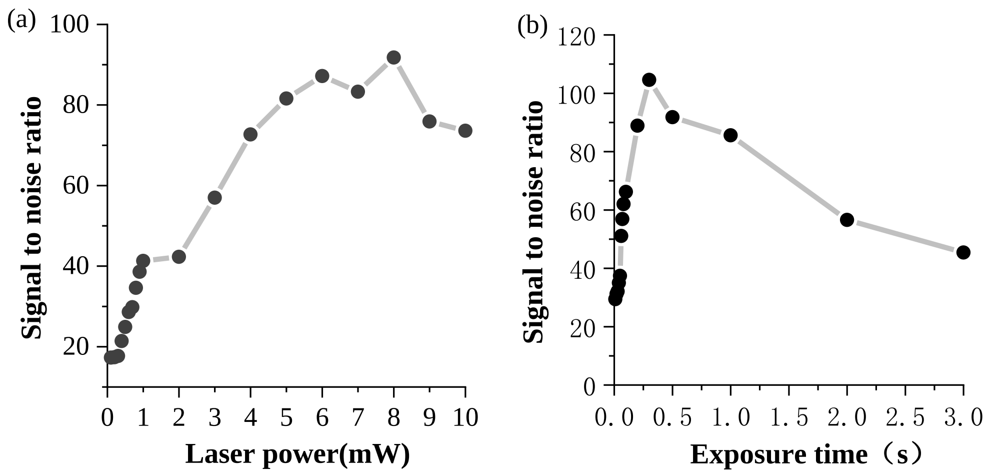

Figure S4 (a) Raman spectral signal-to-noise ratio at different laser powers; (b) Raman spectral signal-to-noise ratios at different exposure times

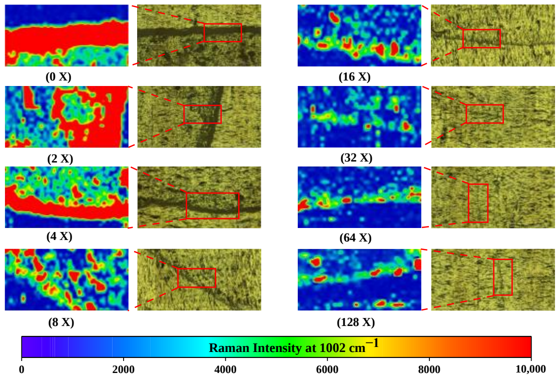

Figure S5 Raman mapping and microscopy images of polystyrene at 474 nm under various dilution factors

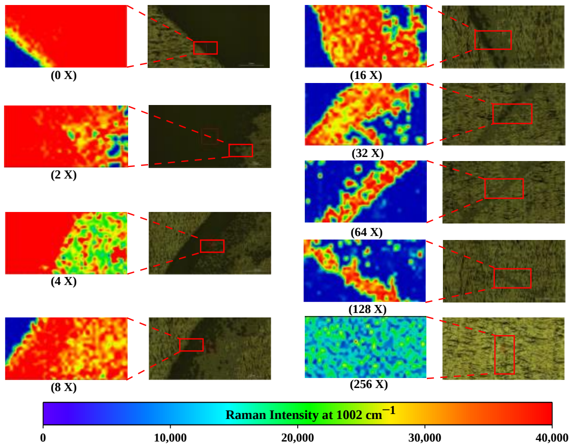

Figure S6 Raman mapping and microscopy images of polystyrene at 1729 nm under various dilution factors

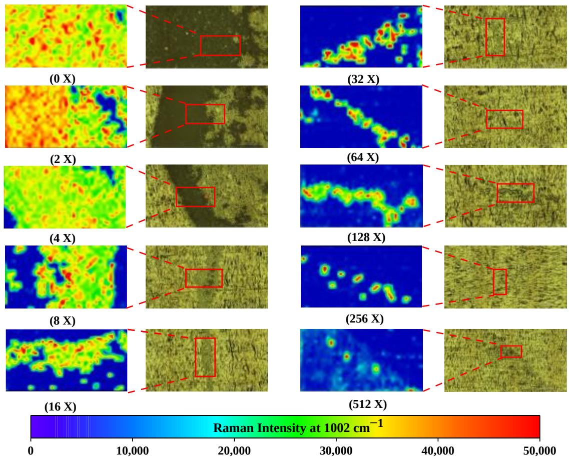

Figure S7 Raman mapping and microscopy images of polystyrene at 2823 nm under various dilution factors

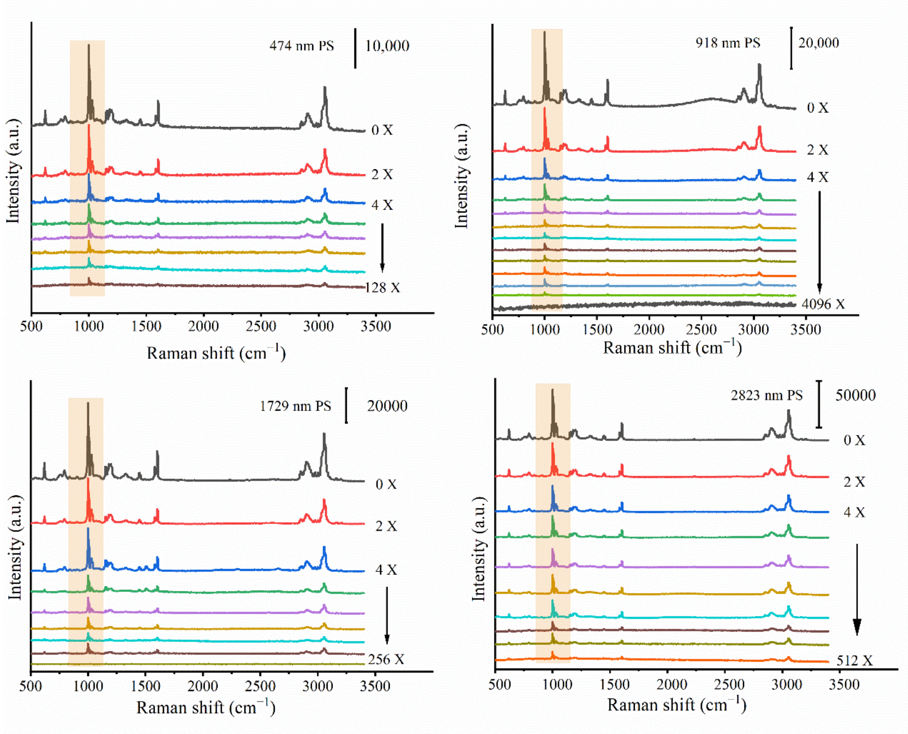

Figure S8 The average Raman spectra of polystyrene (474 nm, 918 nm, 1729 nm, and 2823 nm) at different concentration

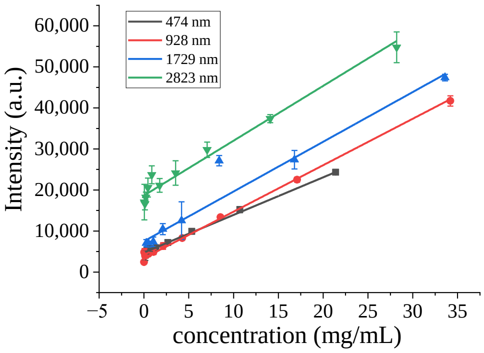

Figure S9 The calibration curves of polystyrene (474 nm, 918 nm, 1729 nm, and 2823 nm) at different concentrations

Table S5 Standard calibration curves and R^2 values for polystyrene at different concentrations

Size(nm)	calibration curves	R^2
474	Y = 915.94X + 4767.08	0.9995
928	Y = 1132.32X + 3394.93	0.9863
1729	Y = 1212.35X + 7501.30	0.9683
2823	Y = 1335.80X + 18653.10	0.9758

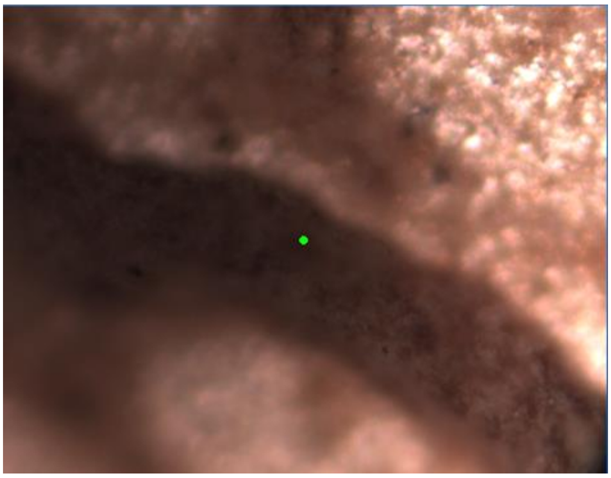

Figure S10 Coffee ring in the spiked flavored yogurt.

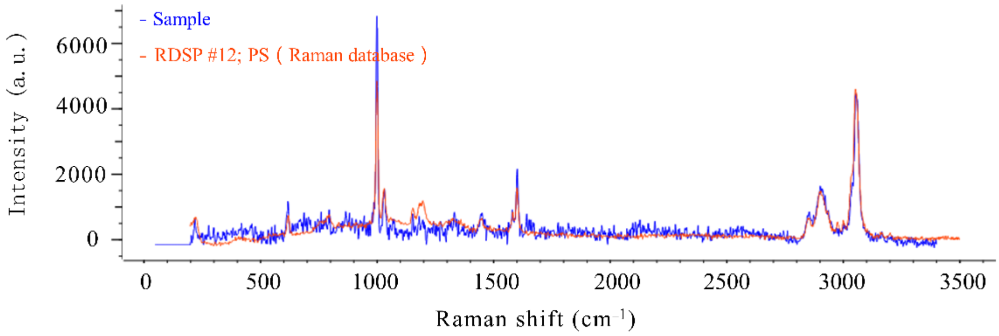

Match score	Database	Serial number	Name	Raman spectrum
79	RDSP	12	PS	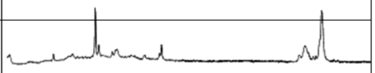

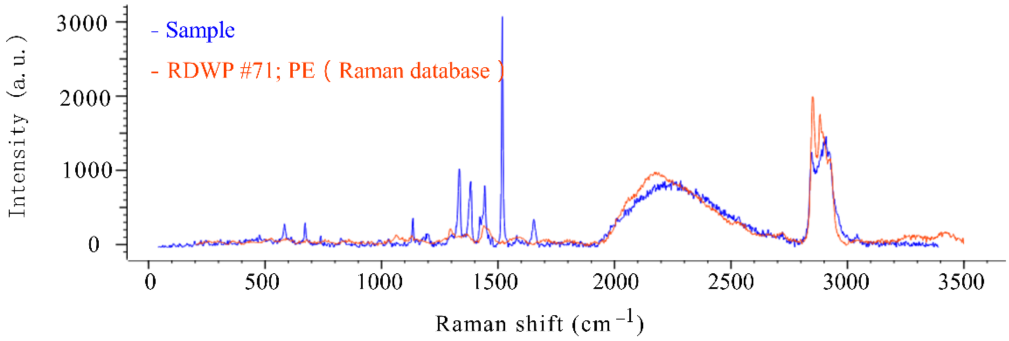

Match score	Database	Serial number	Name	Raman spectrum
74.35	RDWP	71	PE	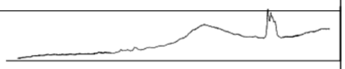

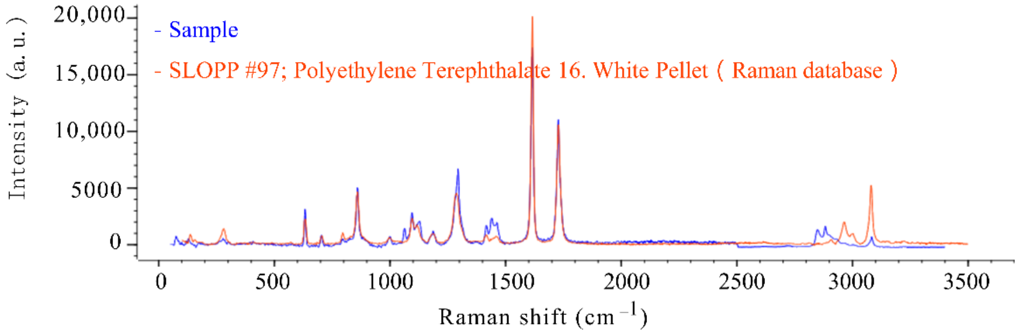

Match score	Database	Serial number	Name	Raman spectrum
76.80	SLOPP	97	Polyethylene Terephthalate 16. White Pellet	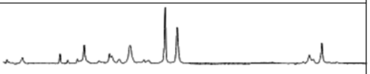

Figure S11 A comparison between obtained spectra and standard polymer spectra.

References

1. Zhang, J.H.; Chen, Z.; Wang, Z.L.; Zhang, W.Y.; Ming, N.B. Preparation of mohodisperse polystyrene spheres in aqueous alcohol system. Mater. Lett. 2003, 57, 4466–4470.

2. Tanaka, K.; Takahashi, Y.; Kuramochi, H.; Osako, M.; Tanaka, S.; Suzuki, G. Preparation of Nanoscale Particles of Five Major Polymers as Potential Standards for the Study of Nanoplastics. Small 2021, 17, 2105781.

3 Nava, V.; Frezzotti, M.L.; Leoni, B. Raman Spectroscopy for the Analysis of Microplastics in Aquatic Systems. Appl. Spectrosc. 2021, 75, 1341–1357.
